# Preconception and Diabetes Information (PADI) App for Women with Pregestational Diabetes: a Feasibility and Acceptability Study

**DOI:** 10.1007/s41666-021-00104-9

**Published:** 2021-08-26

**Authors:** Chidiebere H. Nwolise, Nicola Carey, Jill Shawe

**Affiliations:** 1 Health Services Research Unit, Nuffield Department of Population Health, University of Oxford, L1/16 Richard Doll Building, Old Road Campus, Headington, Oxford, OX3 7LF UK; 2 School of Health Sciences, Faculty of Health & Medical Sciences, University of Surrey, Guildford, UK; 3 School of Nursing & Midwifery, Faculty of Health, University of Plymouth, Plymouth, UK

**Keywords:** Preconception care, Women, Education, Diabetes mellitus, Technology, Smartphone, Mobile applications

## Abstract

Diabetes mellitus increases the risk of adverse maternal and fetal outcomes. Preconception care is vital to minimise complications; however, preconception care service provision is hindered by inadequate knowledge, resources and care fragmentation. Mobile health technology, particularly smartphone apps, could improve preconception care and pregnancy outcomes for women with diabetes. The aim of this study is to co-create a preconception and diabetes information app with healthcare professionals and women with diabetes and explore the feasibility, acceptability and preliminary effects of the app. A mixed-methods study design employing questionnaires and semi-structured interviews was used to assess preliminary outcome estimates (preconception care knowledge, attitudes and behaviours), and user acceptability. Data analysis included thematic analysis, descriptive statistics and non-parametric tests. Improvements were recorded in knowledge and attitudes to preconception care and patient activation measure following the 3-month app usage. Participants found the app acceptable (satisfaction rating was 72%), useful and informative. The app’s usability and usefulness facilitated usage while manual data input and competing priorities were barriers which participants felt could be overcome via personalisation, automation and use of daily reminders. This is the first study to explore the acceptability and feasibility of a preconception and diabetes information app for women with diabetes. Triangulated data suggest that the app has potential to improve preconception care knowledge, attitudes and behaviours. However, in order for women with DM to realise the full potential of the app intervention, particularly improved maternal and fetal outcomes, further development and evaluation is required.

## Introduction

Diabetes mellitus (DM) is a global emergency with significant economic and health impact [1, 2]. Worldwide, 463 million people live with DM, of which 222.9 million are women [1]. The increasing prevalence of DM around the world, and in women of childbearing potential in particular, is concerning as DM increases the risk of complications and adverse outcomes for the mother (e.g. miscarriage, hypertensive disorders and mortality) and fetus (e.g. congenital malformation and perinatal mortality) [3–5]. Globally, £580 billion is spent on DM [1] and healthcare systems, such as the National Health Service (NHS) which spends over £10 billion a year on DM, are struggling to meet increasing costs [1, 2]. Pregnancy-related complications in women with DM contribute to this high healthcare expenditure; for example, a delivery with complications costs the NHS £692 more than one without complications [6]. Preconception care (PCC), educating and supporting women to improve their health behaviours before conception reduce costs by safeguarding the health of women and their babies during and after pregnancy [6–11]. Women with DM who receive PCC experience improved glycaemic control, alongside improved maternal and fetal outcomes [8, 9]. Hence, improving PCC uptake for all women of childbearing age is a major priority of the World Health Organisation (WHO) [12], and the focus of national and international guidelines [3, 10]. Despite this, there is consistent evidence reporting that traditional PCC delivered via face to face encounters in the clinic setting is limited by several factors, i.e. inadequate knowledge, resources and care fragmentation [13–15]; consequently, less than 50% of women with DM have access to PCC around the world [9, 11].

Electronic health (eHealth), accessed through mobile phones, multimedia and the internet, has the potential to improve PCC provision [9]. eHealth plays an instrumental role in improving access to healthcare where resources are scarce, supporting individuals to improve their health and empowering individuals to take a more active role in their healthcare [16, 17]. For example, eHealth via the internet and mobile technology (short message service) has been used to support behaviour change including improving self-efficacy and managing excessive gestational weight gain in indigent women [18–21], optimising blood glucose control and supporting smoking cessation in pregnant women [22, 23] as well as improving nutrition and lifestyle in pregnant women and couples contemplating pregnancy [24]. Similarly, eHealth PCC delivered via multimedia (CD-ROMs, DVDs) and website significantly improved pregnancy outcomes and self-efficacy to seek PCC in women with DM [25–29]. However, the majority of the eHealth PCC studies have used technology with limited scope, thereby excluding many women without access to computers and DVD players [25–28]; smartphone technology has the capacity to make PCC accessible across geographical locations and socio-economic groups [30]. Globally, 3.8 billion people have access to the internet and 3.5 billion (45% of the population) own a smartphone [31, 32]. A recent report on Internet use [32] showed that of the average 6.3 hours spent on the Internet per day, 57% of the time was spent accessing it via smartphones compared with computers or laptops (32%) and other connected devices (11%). Smartphones offer voice and text facility, internet access, geo-positioning systems, high-quality cameras, access to data anywhere and anytime and the capability to support software applications [33–35]. Hence, smartphones are increasingly being used to promote healthy behaviours in people with chronic conditions including DM [36, 37]. Yet, despite the reach and acceptance of smartphones and the internet, very few studies have used eHealth to supplement traditional PCC [25–29]. Consequently, insufficient PCC coverage and subsequent high rates of adverse maternal/fetal outcomes have remained a persistent public health challenge [9, 38–40]. There is therefore a need to leverage smartphone use in the delivery of PCC to women with DM [9, 13, 41].

Mobile Health (mHealth, the medical and public health practice that is supported by mobile devices, e.g. smartphones and apps) presents an opportunity to reach a larger number of women around the world, including those who are less likely to seek PCC and/or engage with healthcare professionals [30, 41, 42]. Smartphones are now considered learning tools due to their capacity to meet educational requirements and significantly improve health outcomes [43, 44]; consequently, smartphone apps have become very popular. In 2017, 3.7 billion health apps were downloaded compared to 1.7 billion in 2013 [45]. Apps have contributed to healthy behavioural changes in several areas including medication adherence, diet control, weight loss, physical activity, lifestyle improvement, smoking cessation and diabetes self-management [27, 46, 47]. Women have expressed a preference for concise healthcare information and personalised support provided via apps [40, 41, 48] and various mHealth apps have been developed to meet the healthcare needs of women including family planning, reproductive health [24, 49] and weight loss [50]. Apps have also emerged as a popular resource to provide information and support to women during pregnancy (e.g. gestational weight gain, physical inactivity, blood glucose management, fetal monitoring, antenatal care attendance, diet and post-delivery support) [48, 51–53]. It is important to note that while there are more apps available for pregnancy than any other areas of health [54, 55], they have to date failed to consider aspects specifically related to preconception care. The scarcity of PCC apps, limitations of face to face PCC service provision and resultant adverse outcomes for women with DM necessitate the development and use of a mHealth app for PCC [9, 41].

Working with healthcare professionals and women with diabetes, a 2-phase study was undertaken to co-create a preconception and Diabetes Information, PADI, app for women with diabetes [41]. This paper reports on the second phase of the study, the primary aim of which is to explore the feasibility, acceptability and preliminary effects of the PADI app on PCC knowledge and attitudes.

### Research Questions

- What are the preliminary estimates of the effect of the PADI app on PCC knowledge, attitudes and behaviours?
- What is the acceptability of the overall app, as measured by satisfaction, usefulness, ease of use and attitudes towards the receipt of the intervention?
- What are the factors that inhibit / facilitate the use of the app?
- What are the suggestions for future app development?

## Methods

### Study Design

A mixed methods approach was used to explore the app’s feasibility and acceptability [56, 57]. The app was developed using a systematic approach in line with the recommendations of the Medical Research Council (MRC) [58], and mHealth development and evaluation frameworks for behaviour change [59–62]. The following two stages have been completed: development (gaining an in-depth understanding of the target population, developing a prototype, gathering feedback and building an initial intervention) and feasibility and acceptability (conducting a small-scale evaluation to test potential efficacy, assessing usability and satisfaction, and conducting interviews to understand user experience).

### Stage 1 Development of the Preconception and Diabetes Information App

The Preconception and Diabetes Information (PADI) app, co-created with healthcare professionals, women with DM and a mobile app development company (Netsells), [57] was designed to improve knowledge of PCC and pregnancy planning, positively influence attitudes towards seeking PCC and improve patients’ activation (knowledge and confidence for self-management of health). The app combines pregnancy planning information with diabetes monitoring functionality so that women would not need to use different apps for diabetes and preconception care, but would be able to use the PADI app for both functions. The iterative process of designing, developing and piloting the PADI app is reported in detail elsewhere [41, 57]. In summary, following input from healthcare professionals and women with DM, the app design was discussed with Netsells (the app developer) and an initial prototype was created, tested in-house for optimal functionality and then released for further testing. The PADI app prototype was pilot-tested for 14 days by a selection of participants (healthcare professionals, women with DM, researchers and members of the public). The piloting comprised two cycles of feedback and resulted in changes to the information pages, blood glucose diary and graphical display of blood glucose readings.

The final PADI app comprised of four components:

- Information on planning for pregnancy: this feature was drafted in line with the National Institute for Clinical Excellence (NICE) preconception care (PCC) guidelines [3]. A mix of textual information, videos and uniform resource locators (URLs, which users can click on to view more information on a specific topic from reliable sources including NICE, Diabetes UK, womenwithdiabetes.net and Family Planning Association) were used to provide information on various aspects of preparing for pregnancy and what to expect during pregnancy and delivery.
- Blood glucose diary with data visualisation: this feature enables the recording of blood glucose reading throughout the day; users select the time of day from a drop-down menu, e.g. before lunch. All entries are displayed in order from the oldest to the most recent entry.
- Reminder to take blood glucose reading: These are set when a new reading is added. A notification is sent to the user’s phone at their set reminder time. Users have the option to deactivate set reminders by toggling the switch.
- A progress functionality to help monitor blood glucose trends: The entered blood glucose readings are broken down into daily averages and represented in a graph and users can choose how they would like their progress displayed, e.g. today, past seven days or 30 days.

### Stage 2 Feasibility and Acceptability

**Fig. 1:**
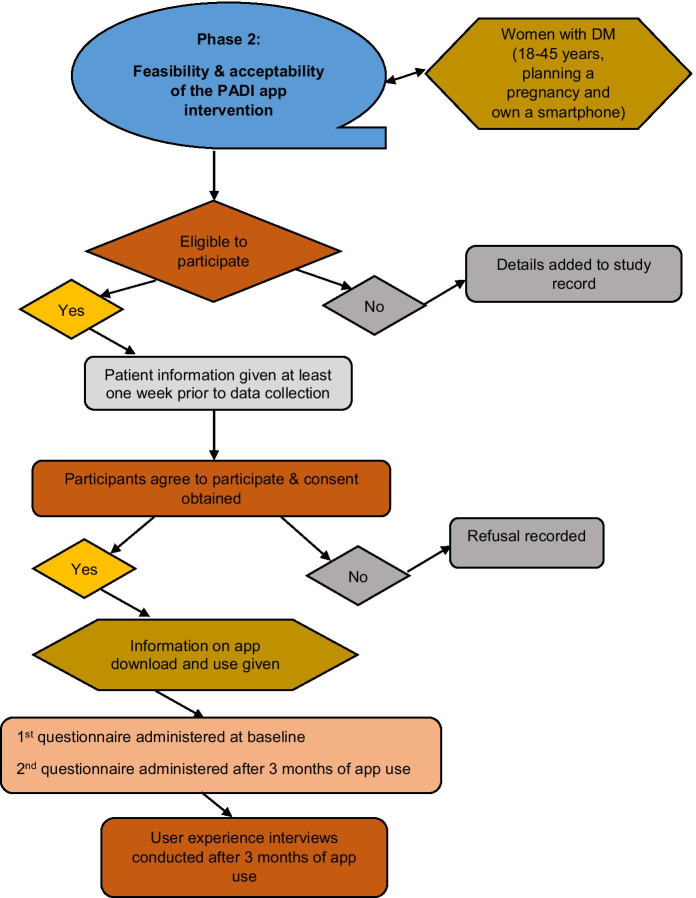
Sequence of activities in phase 2

In line with the Medical Research Council (MRC) framework for complex interventions, mHealth app development frameworks and previous health app studies [46, 58–62], a prospective pre- and post-intervention study was undertaken comprising patient questionnaires (baseline and after 3 months of app use) and semi-structured interviews (Figure 1). Rather than providing evidence of statistical significance, feasibility studies, incorporating quantitative and qualitative methods, provide preliminary data for the primary outcome measure, support exploration of the intervention’s acceptability and help determine the success of future trials [58, 63].

Participants who met the inclusion criteria and provided written informed consent were enrolled into the study. A link to the free app was provided and participants were asked to download the PADI app onto their smartphones from iTunes and Google Play stores. Participants recruited from diabetes clinics (*n* = 2) received a face-to-face demonstration of the key components and functionality of the PADI app, while the same information was communicated to those recruited via Twitter (*n* = 15) remotely (via email and telephone). Participants were encouraged to use the PADI app for 5 minutes a day for a period of 3 months for PCC and self-monitoring of blood glucose levels, in line with previous eHealth feasibility studies [25, 46, 62].

### Ethical Review

Favourable ethnical opinion was obtained from the United Kingdom National Research Ethics Service (UK NRES) Committee East Midlands-Derby, REC reference 15/EM/0358, IRAS project ID: 178530; the University of Surrey Ethics Committee and the Research Governance Committee of two National Health Service (NHS) hospitals.

### Sampling, Recruitment and Consent

A convenience sample of 17 women of reproductive age (18–45 years) with pregestational DM, who were planning a pregnancy in the next 5 years or wanted children at some point in the future, and who owned an iOS or Android smartphone, were recruited into the study. The sample size was informed by previous small-scale mHealth feasibility and pilot studies [46, 60, 62–66] and a feasibility study guideline [67]. The target sample for this phase was (n = 12), a total of (n = 17) participants with type 1 and type 2 DM were recruited to allow for loss due to follow up.

Participants were initially recruited from the outpatients’ diabetes clinics of two National Health Service (NHS) hospitals in the South of England; however, due to delays experienced in recruiting women with DM from healthcare settings, participants were subsequently recruited via social media (i.e. twitter). A tweet was sent out via the University and Department twitter account as well as by other researchers and organisations involved in diabetes research and with access to women with DM, e.g. Diabetes UK, Women with Diabetes and the College Diabetes Network (CDN), inviting eligible participants to contact the study researcher if they were interested in participating in the study. Recruitment took place from December 2016 to March 2017. Informed written consent was obtained prior to data collection.

### Outcome Measures

i) Knowledge of preconception care
ii) Patient activation measure (PAM)
iii) Attitudinal change to PCC

Data were collected to provide preliminary outcome estimates, reflecting women’s experience and satisfaction with the PADI app. As such, there was no primary outcome measure [68].

### Data Collection

Data collection followed a sequential approach. Questionnaire data was collected at baseline (from *n* = 17 participants) and after 3 months of using the App (from *n* = 11 participants who completed the study); semi-structured interviews on a subsection of the sample (*n* = 6) were used to explore the acceptability of the PADI app.

#### Pre and post-intervention questionnaire

A self-administered questionnaire informed by previously validated instruments: an abridged reproductive health attitude and behaviour (RHAB) instrument [69], preconception care knowledge (K) instrument [28] and patient activation measure (PAM) [70] was developed. A woman with DM and a healthcare professional piloted the questionnaire and commented on length and ease of comprehension; based on comments, no changes were made to the questionnaire. Details of data collected, timing, items and instruments are presented in Appendix Table 5.

#### Post-intervention interviews

Participants comprised of (*n* = 6) women with DM who participated in the 3-month app intervention. Semi-structured interviews via the telephone, lasting 20–30 minutes, were undertaken at the end of the study with all six intervention participants who consented to being interviewed [71]. This sample size is considered adequate to gain an in-depth understanding of user experiences and usability issues [72, 73]. An interview schedule, informed by the literature [74, 75] was used to guide the interview, and explore participants’ views and experiences of using the PADI app. The interview schedule was piloted with a woman with DM and healthcare professional, and found to be straightforward. The semi-structured interview guide has been published elsewhere [57]. These evaluative interviews were used to ascertain the app’s usability, acceptability, benefits, challenges, usefulness and areas in need of improvement. These interviews were also used to provide insight into participants’ engagement with the app. Data collection was completed in July, 2017.

### Data Analysis

Questionnaire data were summarised using descriptive statistics and compared to explore preliminary estimates (RHAB, PCC knowledge and PAM) using Wilcoxon-signed rank test. The effect size of the measures was reported using Cohen’s criteria [76, 77] and internal consistency was measured using Cronbach’s coefficient alpha [76]. Satisfaction with the app was rated using a simple visual scale, respondents rated the app from 0 to 100 [where 0 = not satisfied at all and 100 = completely satisfied]. Data were analysed using SPSS Statistics version 25.0 software (IBM Corporation).

Interviews were digitally recorded, transcribed, anonymised and checked for accuracy. Qualitative data were imported into NVivo 11 software (QSR International) and analysed using thematic analysis as described by Braun and Clarke [78]. Transcripts were read thoroughly and coded by one researcher (CHN), transcripts were reread to ensure that no codes had been missed and the themes aligned with the data. The themes generated were then discussed with the other two researchers (NC and JS) until consensus was achieved.

Questionnaire and interview data were subsequently triangulated in order to provide a more holistic interpretation of the data and maximise credibility of the findings. Triangulation facilitates generation of thick description, enhances rigour of a research study and increases confidence in the overall analysis [79].

## Findings

### Recruitment and Retention of Participants

Thirty-eight women with type 1 and type 2 DM expressed an interest in joining the study; 35 responded to a twitter advert and 3 responded to a face-to-face invitation. After receiving further study information, 19 (50%) women declined participation, while two (5%) were excluded as they had completed their families and were not eligible for inclusion. Seventeen (45%) women agreed to participate, provided written consent and were enrolled into the study. The pre-intervention questionnaire was sent to all 17 women before they were given the link to the app download. Post-intervention questionnaire was received from 11 (65%) participants. The reasons given for leaving the study included work/time constraint (*n* = 1) and no longer planning a pregnancy (*n* = 1). Four other participants did not give a reason and could not be contacted despite three reminders. The attrition rate was estimated at (6/17) 35%.

### Demographic Profile

Participants were geographically dispersed across the UK (*n* = 13, 76%) and North America (*n* = 4, 24%). All participants were white, with the majority married or living with a partner (*n* = 11, 65%). Age ranged from 20 to 43 years (range = 23). The majority of participants (*n* = 16, 94%) had type 1 DM, were employed (*n* = 14, 82%) and had a first or higher academic degree (*n* = 11, 65%). Most women (*n* = 12, 70%) reported plans for a pregnancy in the near future (i.e. less than 1 year or in 1–5 years) and previously receiving PCC advice (*n* = 15, 88%). The demographic characteristics of the pre-intervention (*n* = 17) and post-intervention (*n* = 11) study participants are shown in Table 1. The demographic profile of the (*n* = 6) participants who took part in the semi-structured interviews is shown in Table 2.

**Table 1:** Pre-intervention and post-intervention demographic profile

	Pre-intervention	Post-intervention	Non-responders*
Characteristic	n	n	n
Total sample	17	11	6
Demographic characteristics			
Age (years)	31.3 ± 6.7	31 ± 6.6	31.83 ± 7.5
Marital status			
Married/living with partner Single (never married)	11(65%) 6 (35%)	8 (47.1) 3 (17.6)	3 (17.6) 3 (17.6)
Diabetes type			
Type 1 Type 2	16 (94%) 1 (6%)	10 (58.8) 1 (5.9)	6 (35.3) 0 (0)
Diabetes duration			
Less than 1 year Over 5 years	1 (6%) 16 (94%)	1 (5.9) 10 (58.8)	0 (0) 6 (35.3)
Race or ethnic group			
White British Irish Any other white background	9 (53%) 2 (12%) 6 (35%)	4 (23.5) 2 (11.8) 5 (29.4**)**	5 (29.4) 0 (0) 1 (5.9)
Geographical location			
United Kingdom America Canada	13 (76%) 2 (12%) 2 (12%)	– – –	– – –
Highest educational qualification			
Higher degree (M.Sc or PhD) First degree (B.Sc) Other diplomas A / AS / S levels Other academic qualifications None of these qualifications	6 (35%) 5 (30%) 2 (12%) 2 (12%) 1 (6%) 1 (6%)	6 (35.3) 2 (11.8) 1 (5.9) 2 (11.8) 0 (0) 0 (0)	0 (0) 3 (17.6) 1 (5.9) 0 (0) 1 (5.9) 1 (5.9)
Employment status			
Employed full-time/part time Full-time homemaker Student	14 (82%) 1 (6%) 2 (12%)	9 (52.9) 1 (5.9) 1 (5.9)	5 (29.4) 0 (0) 1 (5.9)
Currently considering or planning to have children			
In less than 1 year In 1–5 years Over 5 years Do not know/unsure	6 (35%) 6 (35%) 3 (18%) 2 (12%)	6 (35.3) 2 (11.8) 2 (11.8) 1 (5.9)	0 (0) 4 (23.5) 1 (5.9) 1 (5.9)
Previously had a pregnancy that ended in:			
Miscarriage Ectopic pregnancy Other (e.g. termination or preterm birth)	1 (6%) 1 (6%) 2 (12%)	– – –	– – –

*Participants who dropped out and did not complete the study

**Table 2:** Interview participants’ descriptive table

No	Identifier	Age (years)	Diabetes mellitus type & duration (years)	Educational qualification	Planning a pregnancy
1.	P1	24	Type 1 (> 5)	Higher degree (M.Sc or PhD)	> 5 years
2.	P2	36	Type 1 (> 5)	First degree	< 1 year
3.	P3	33	Type 1 (> 5)	Higher degree (M.Sc or PhD)	< 1 year
4.	P4	32	Type 1 (> 5)	Higher degree (M.Sc or PhD)	< 1 year
5.	P5	20	Type 1 (> 5)	A levels	> 5 years
6.	P6	39	Type 1 (> 5)	First degree	< 1 year

### Questionnaire and Interview Results

The results of the pre- and post-intervention questionnaires and individual semi-structured interviews are presented collectively. Analysis identified three main themes: (1) effect of the PADI app on PCC knowledge and behaviour, (2) satisfaction and engagement with the PADI app and (3) future development of the PADI app; all themes had sub-themes.

### Effect of the PADI App on PCC Knowledge and Behaviour

#### Knowledge of Preconception Care

Generally, the PADI app was viewed by those participating in interviews as an ideal means of providing PCC because of its accessibility and portability. Participants considered the app to be a comprehensive and informative source of PCC, which incorporated self-management of blood glucose (SMBG) functions, and felt that the PADI app helped to improve understanding of pregnancy planning and pregnancy-related risks.

*I like it. I’ve carried around a book but I either don’t have a pen or my handbag is too small for it or I’ve left it at home and it’s very rare that I leave my phone behind … I can … always put [blood glucose readings] in … and I just like the fact that it’s like having all the information in a book but in a tiny place on your phone. [P6, 39 years]*

*Just greater emphasis on blood sugar control, which I knew was an issue but knowing specifically how important it is from the app, it’s definitely made a big impact and knowing what research resources are available to me. [P5, 20 years]*

Evidence of the effect of the PADI app on PCC knowledge is supported by questionnaire data (Table 3). At baseline (pre-intervention), participants achieved scores of 100% for four pregnancy planning items compared to post-intervention where scores of 100% were achieved for six items including preconception HbA1c and safety of over-the-counter medications. Pre-intervention pregnancy-related risk scores were 100% for five items pre- and post-intervention; knowledge of risk of miscarriage also improved pre- to post-intervention. Following the PADI app intervention, a statistically significant increase was observed in knowledge of pregnancy planning (*P* = .03) but not pregnancy-related risks (*P* = 0.71).

**Table 3:** Knowledge of PCC: correct answers pre- and post-intervention

Knowledge statement	Correct response	Pre-intervention no (%) of participants correct (*n* = 11)	Post- intervention no (%) of participants correct (*n* = 11)
Knowledge of pregnancy planning			
1. Women who are planning a pregnancy should discuss medication use with a healthcare provider	T	11 (100)	11 (100)
2. Women who are planning a pregnancy should stop smoking	T	11 (100)	11 (100)
3. Before becoming pregnant, ideally your HbA1c should be below 6.5% (48.0 mmol/mol)	T	10 (91)	11 (100)
4. Women with diabetes cannot use hormonal contraception	F	11 (100)	11 (100)
5. Women with diabetes have very limited choices of contraception	F	11 (100)	11 (100)
6. Women with diabetes should take folic acid daily when planning a pregnancy	T	9 (82)	10 (91)
7. Women who are planning a pregnancy should stop drinking alcohol	T	9 (82)	10 (91)
8. All over the counter drugs are safe and can be taken by women with diabetes who are planning a pregnancy	F	8 (73)	11 (100)
9. If you have Type 2 diabetes and are planning to become pregnant you may need to change from tablets to injections of insulin	T	6 (55)	6 (55)
10. Women with diabetes should take the same amount of folic acid as all other women planning a pregnancy	F	4 (36)	8 (73)
11. All insulin are suitable for use during pregnancy	F	1 (9)	4 (36)
Knowledge of pregnancy-related risks			
12. High blood glucose levels during pregnancy do not increase the risk of problems for the mother	F	11 (100)	11 (100)
13. High blood glucose levels during pregnancy do not increase the risk of problems for the baby	F	11 (100)	11 (100)
14. Women with diabetes have little control over the health of their baby	F	11 (100)	11 (100)
15. Chances of a woman having a healthy baby increase as she improves her health prior to conception	T	11 (100)	10 (91)
16. Women with diabetes can have a healthy baby	T	11 (100)	11 (100)
17. Blood glucose levels before pregnancy can affect the health of the baby	T	10 (91)	10 (91)
18. Women with diabetes have an increased risk of having a large baby making delivery more difficult	T	10 (91)	11 (100)
19. Women with diabetes do not have an increased risk of having a baby with birth defects	F	8 (73)	7 (64)
20. Women with diabetes have an increased risk of miscarriage	T	4 (36)	9 (82)

#### Behavioural Effects of the PADI App

Participants reported that improved knowledge in turn reduced any previous anxieties that they had about planning and having a healthy pregnancy. The app intervention enabled women to recognise the importance of involving their healthcare team in pregnancy planning. For women planning a pregnancy in less than one year, the app usage facilitated PCC discussions with their healthcare team while those planning to have a baby in future, would seek PCC and discuss pregnancy intention prior to conception.

*I think when I do decide I would like to become pregnant I definitely would seek preconception care. I’d probably go to my GP and look for other resources. Yes, I think the app and being part of this study just opened my mind as to what care is out there and where I can search for more care and more support. [P1, 24 years]*

*I did speak to the diabetes team and had the HbA1c taken and then we had a bit of discussion about it … Taking the extra folic acid … it’s one of the main things that I found out about … it was interesting to know that you need to take more than you generally hear about. It wasn’t something that I had been aware of at all … I was actually showing that to my doctor and said, “Well actually it says that we need the 5mg rather than the normal [400µg]” so I’ve had that prescribed now. [P6, 39 years]*

The PADI app intervention also had a positive effect on all five RHAB constructs included in this study (Table 4). That is, perceived benefits of PCC, self-efficacy and outcome expectations increased while perceived susceptibility and barriers decreased pre to post-intervention; however, these changes were not statistically significant. Similar to previous studies, [25, 28] Cronbach alpha coefficient for the RHAB constructs ranged from 0.4 to 0.7 (pre-intervention) and 0.6 to 0.9 (post-intervention).

**Table 4:** Beliefs and attitudes associated with preventing an unplanned pregnancy and seeking PCC pre- and post-app intervention

Questionnaire measures	Possible scale range	Participants’ pre-intervention attitudes (*n* = 11)		Participants’ post-intervention attitudes (*n* = 11)		Mean difference	P*	Effect size	Cronbach alpha pre and post-intervention
		Mean & standard deviation	Median	Mean & standard deviation	Median				
Susceptibility	4–20	11.09 ± 2.98	10	9.18 ± 2.60	9	− 1.9	0.07	0.38	0.6, 0.6
Benefit	2–10	9.18 ± 1.08	10	9.55 ± .93	10	0.37	0.29	0.23	0.5, 0.7
Barriers	2–10	5.09 ± 2.13	5	4.27 ± 2.24	3	− 0.82	0.32	0.21	0.5, 0.9
Self-efficacy	3–15	11.64 ± 1.89	12	12.55 ± 1.81	12	0.91	0.18	0.29	0.4, 0.8
Outcome expectations	4–20	13.73 ± 3.88	16	15.1 ± 3.72	13	1.37	0.75	0.07	0.7, 0.6

*Pre-intervention scores versus post-intervention score by Wilcoxon signed rank test

In addition, a positive effect was observed in PAM levels. At baseline (pre-intervention), 11 participants (64.7%) were at level 4, four (23.5%) at level 3, two (11.8%) at level 2 and none were at the lowest level of activation (level 1). Of the (*n* = 11) participants who completed the post-intervention questionnaire, two (18.2%) had increased their PAM levels from 2 to 4, three (27.3%) from 3 to 4 and five (45.5%) who were already at level 4 had increased PAM scores post-intervention; only one participant remained at the same level of activation (level 3). The PAM score increased significantly (*P* = 0.006) following the intervention with a large effect size (*r* = 0.58). The Cronbach alpha scores for the 13-item PAM were high pre- to post-intervention (0.86, 0.87).

### *Satisfaction and Engagement with the PADI App*

All (*n* = 11) participants who completed the post-intervention questionnaire rated the PADI app in terms of their overall satisfaction with the app and its functionalities. The average app rating based on the scores of all (*n* = 11) participants = 71.8 (range 25–90). A small number of participants (*n* = 2, 18%) rated the app ≤ 50 while the majority (*n* = 9, 82%) gave it a rating above 60. Reasons for the ratings were not provided; however, interview data showed that while most of the participants were pleased with the overall app and its functionalities, the limitations of the blood glucose diary may have affected the rating. Furthermore, all interviewed participants reported that they had used the PADI app to access PCC information, but majority reported using it periodically to record blood glucose levels.

*I think it’s a brilliant app … and anyone I do know I will be recommending it to them. [P4, 32 years]*

*I read everything that you wrote about like the planning … but like the blood glucose monitoring, when you have it everywhere else, to put it somewhere else was just not… I just did not keep up with it. [P13, 33 years]*

#### Facilitators

##### Usability and Usefulness

Participants reported finding the PADI app straightforward to download and install onto their mobile phones. Participants further reported that it was easy to navigate the different sections of the app and generally liked the user interface (UI), noting that it was simple, attractive and intuitive. As well as the informational content, the app also contained links to additional resources which participants found beneficial.

*Quite easy to use. It looks nice, it was quite self-explanatory. [P6, 39 years]*

*The information was very good and the fact that it’s got links to things was also very good. Rather than having to search it for yourself, you could press the link and go straight there … Being able to log in to forums about pregnant diabetics … and be able to hear about other people in the same situations … It’s some of the most useful things I’ve seen [P6, 39 years]*

#### Barriers

##### Self-monitoring and Competing Priorities

Participants wanted simple and fast functionality with minimum effort on their part and although the blood glucose diary calculated average blood glucose levels, had a graph that displayed trends and a reminder built into the app, the manual entry of blood glucose readings was reported to be time-consuming. The diary also lacked the facility to email or download readings, estimate HbA1c or log other pertinent information such as insulin.

*I’m using an insulin pump and my own blood glucose meter does give me a lot of the information and the downloads and the working out of what my average levels are, which times of the day I’m mainly maybe at risk of having high blood sugar … so I have got that already, so I didn’t really feel that the app gave me any additional benefits. [P2, 36 years].*

Other aspects in participants’ lives also influenced their engagement with the app. The main commitments such as work and diabetes management interfered with participants’ use of the PADI app and caused them to deviate from using it regularly.

*It’s very hard as a diabetic when you have to wake up in the morning and you have to remember to do your blood and to do your insulin … I think I would have used it a lot more if my alarm hadn’t have been going off and I hadn’t been rushing out the door and then just work and then suddenly it was the next day. [P4, 32 years]*

##### Not Planning a Pregnancy and the Role of Memory/Time

Women who were not planning a pregnancy in the short-term such as in the next one year reported not using the app regularly because they did not perceive accessing the information via the PADI app as a priority during the study intervention period. In addition to not planning a pregnancy, participants had to deal with a lot of things on a daily basis, including life in general and the complexity of managing a long-term condition, which caused them to forget to use the app.

*I’m also not planning pregnancy at the moment, so the information wasn’t really on my mind all the time. [P11, 24 years].*

*I wasn’t actually trying to get pregnant but because of all the other things in life I forgot. [P14, 32 years]*

Participants reported that although the app could easily fit into their day-to-day routine as it did not take up much time, they still did not use the diary to record blood glucose levels immediately after taking their readings because they forgot or did not have time. Time constraint and memory therefore affected the frequency of use.

*It would be quite easy to fit in if I made sure that I did it … I think if I set my mind to it … it would only be another minute when I wasn’t taking my readings … sometimes I forgot and I’d think “oh I don’t have the time now, I’ll do it later on.” [P16, 39 years]*

### Future Development of the Intervention

#### Informational Content

Participants emphasised the usefulness of the PADI app information but acknowledged the lack of clear direction and information about pregnancy from healthcare professionals and online resources, and wanted the app to help fill the gap regarding what women with DM should do when they first find out that they are pregnant. Hence, they wanted more information on diabetes and pregnancy.

*I mean, it’s okay as it is but … I think more would be even better. Someone downloading this app wants as much information as they can, are nervous about their diabetes and their pregnancy and so I think more is good. [P3, 33 years]*

#### Blood Glucose Diary

Most participants strongly recommended that the diary be enhanced and refined in order to reduce user burden and increase efficiency. These recommendations pertained to automation of the diary, adding a free text option to assist women in making sense of a high or low blood glucose reading, and logging pertinent information such as insulin, diet, exercise or sleep.

*It would be brilliant if you could link it into a sugar monitor or from a Bluetooth sort of monitor or something so that it could automatically go in. That would make it much easier, because that would save so much time. [P6, 39 years]*

*I think potentially having more ability to log different circumstances within your glucose diary, being able to log information. [P1, 24 years]*

#### General App Features

Participants recognised the importance of personalising the app or tailoring it to better meet the needs of women at different stages of the pregnancy planning journey. They also felt that women would benefit from periodic motivational messages and daily reminders to record blood glucose levels.

*Perhaps if you put in how far in the future you want to be pregnant and it brought up more relevant things, that could be a very useful thing, so like if I want to get pregnant in six months then maybe you need to think about folic acid … or now’s the time to go and get your HbA1c. [P6, 39 years]*

*So my recommendation would be that it should have in the morning a reminder or a notification or some kind of alert to make you think about it, so to enter your blood glucose levels. I think that would be really helpful. [P4, 32 years]*

## Discussion

To our knowledge, this is the first study to co-design a preconception and diabetes information (PADI) app for women with DM and examine its feasibility, acceptability and preliminary intervention estimates. PCC education has been recommended as an effective strategy to promote PCC knowledge and behaviour [3, 10, 12] and the study findings showed that women’s overall knowledge of pregnancy planning and risks, and understanding of diabetes and pregnancy improved after the PADI educational app intervention. These findings are consistent with previous studies that have used eHealth technology to raise awareness of PCC and promote behaviour change in women with DM [25–29]. In line with the literature, [80] participants reported feeling supported by the PADI app intervention, and experiencing reduced anxiety along with improved confidence to plan a pregnancy and seek PCC. The PADI app intervention may have also contributed to an increase in patient activation measure (PAM), which has a direct relationship with adoption and sustenance of healthy preventive behaviours, such as blood glucose optimisation [81]. Preliminary results indicated that the app has the capacity to address the key determinants of PCC behaviour change in women with DM specifically related to knowledge, attitudes and self-efficacy [82].

Participants reported involving or being willing to involve healthcare professionals in pregnancy planning and preconception health checks. The ability to make reproductive health decisions and initiate discussions with healthcare professionals are key modifying factors that affect women’s intention to seek PCC and plan a pregnancy [26, 83]. According to the Expanded Health Belief Model, a social cognitive model used to predict health behaviours, these modifying factors contribute to behaviour change [83]. This argument is supported by Kalua and Nyasulu [84], who note that although knowledge influences attitudes, it does not guarantee a desirable behaviour; other modifying factors that enable an individual to engage in healthy behaviours have to be present. A literature review on the use of technology to empower patients found that health literacy, remote access to healthcare and self-management mechanisms were the most valued means of achieving patient empowerment and behaviour change [85, 86]. Researchers [24, 43] argue that PCC education provided via mobile technology can improve both health literacy and behaviours. Several educational mhealth interventions have reported success in improving reproductive health knowledge and changing women’s behaviour including Smarter pregnancy which improved folic acid intake and lifestyle in pre-pregnant women [24, 43], Text4two reduced gestational weight gain in pregnant women and improved self-monitoring and lifestyle [44], and Waiting Room app improved knowledge of contraception and family planning [49]. Chen and Mangone [87] also highlighted the role of apps in preventing unplanned pregnancy in young women; however, these are not focussed on diabetes. The improved interaction between women with DM and their healthcare professionals in this study, which mirrors the results of another eHealth PCC intervention for young women with DM in America [26], demonstrates that by empowering women to initiate PCC discussions, the PADI app could improve PCC uptake and pregnancy outcomes.

Evidence from this study suggests that the PADI app could be used to overcome traditional problems in PCC, providing 24 h access to consistent and comprehensive PCC information and advice from any geographical location. Although several eHealth technologies have been used to provide PCC to women with DM, they offer limited scope to women who are increasingly using the internet and smartphone apps to access healthcare information [9, 41, 54, 74, 87, 88]. In line with the literature, [89] it appears that participants accepted the app as they found it simple, informative and useful. The acceptability of the PADI app may have contributed to the high satisfaction rating and achievement of preliminary intervention effects (i.e. improved knowledge, attitudes and self-management capacity). Evidence shows that participants who view an intervention as unacceptable tend to withdraw from it while those who accept the intervention rate it favourably, use it and experience its beneficial effects [90]. Furthermore, the involvement of service users and healthcare professionals in the intervention development may have enhanced the intervention’s acceptability by ensuring that the app was consistent with, and responsive to participants’ preferences and needs [90, 91]. The successful use of the PADI app to provide uninterrupted access to PCC across three countries demonstrates its potential ability to meet the PCC needs of women with DM around the world. This is particularly important given the severe adverse effects of inadequate PCC.

The feasibility testing of the app provided the opportunity to identify aspects that would benefit from further development, further enhancing its acceptability and usage in the future. Guidelines for developing mHealth interventions for behaviour change [59–61] recommend testing the innovations early, in order to analyse usage behaviour, understand user experiences and highlight any design faults or limitations. In line with the literature [92–94], our findings suggest that despite acceptability and a high user satisfaction rating, user engagement with the app was moderate and affected by several factors including usability, competing priorities, pregnancy intention, memory, time and manual data input. Although mHealth apps are viewed as helpful, useful and convenient, continued usage often lags behind and drop-out rates are high [95]. Hence, long-term engagement with apps is of concern for researchers involved in health app development because insufficient engagement often leads to attrition of a substantial proportion of users who drop out prematurely or stop using the app [96]. Moreover, high drop-out rates have been associated with health app use; for example, a retrospective study of a dietary self-monitoring mobile app found that less than 3% of 190,000 people who downloaded the app used it actively for over one week [97]. Furthermore, compared to communication apps which make up 49.5% of app launches, the proportion of health app use is only about 0.26% of all app launches. Evidence [98] also shows that service users prefer to engage with health apps periodically; however, increased engagement via the repeated use of technology, e.g. apps and websites, is associated with improved health outcomes [99–101]. Factors contributing to reduced motivation to use health apps include lack of personalisation, ability to share information, integration with glucometer or insulin pump, timely self-management information and feedback that targets individual needs [102]. People with DM in particular prefer automated and intelligent systems that could take over their tasks in order to provide relief from their self-management responsibilities [102]. Thus, as highlighted by participants in this study and relevant literature [103, 104] automation, improved self-monitoring capacity, use of notifications and personalisation, may be vital for sustaining engagement and improving maternal and fetal outcomes.

### Strengths and Limitations

Studies using smartphones to provide PCC to women with DM are scarce; this study has developed and tested the feasibility and acceptability of a preconception and diabetes information (PADI) app for women with DM. The preliminary findings suggest that the PADI app has the potential to improve knowledge and attitudes to PCC and PAM; however, as a feasibility study, it is important to note that the aim was not to detect statistical changes. The small size, non-random sample and quasi-experimental study design used may affect the internal validity of the study; hence, the findings are indicative and should be interpreted with caution. Comprehensive log of activity data would also be important to include in a future study as this would enable quantitative analysis of app usage behaviour. Participants were mostly recruited via social media and it is possible that the more motivated and highly-educated women responded to the invitation to participate in this study. In a future study, it would be interesting to see if there were differing results across educational levels. Furthermore, recruitment in this study was targeted at women who were planning a pregnancy in the next five years or wanted children at some point in the future; however, because some users were not planning a pregnancy imminently, engagement with the app may have been affected and for future studies a more targeted recruitment strategy needs consideration.

## Conclusion

mHealth is increasingly being used to improve access to healthcare around the world. The study findings demonstrate that a smartphone application is an acceptable way of providing PCC to women with DM and could help improve current shortfalls in PCC service provision. As such, it provides a unique contribution to knowledge, which can influence future PCC service delivery and lead to healthy outcomes of pregnancy for women with DM. Preliminary results indicate that the PADI app may help improve knowledge, attitudes and behaviours towards PCC, which has positive implications for pregnancy planning and PCC uptake. However, in order for women with DM to realise the full potential of the PADI app intervention particularly improved maternal and fetal outcomes, further development and evaluation is required.

## Appendix

**Table 5:** Summary of data collection arrangements and instruments

Category of data	Method of data collection	Timing of collection	Items and instruments
1) Patient characteristics	Pre-intervention questionnaire	At baseline	*Socio-demographics*: age, diabetes type and duration; pregnancy intention; employment; education; ethnicity; previous adverse outcome.
2) Patient activation measure	Pre- and post-intervention questionnaire	At baseline and after 3 months of using the app	20 (5-point Likert Scale) questions exploring reproductive health, attitudes and behaviour (RHAB) [69]. The RHAB is a validated theory-based instrument, with acceptable levels of validity and reliability (Cronbach’s alpha = 0.65–0.83), that measures the decision-making factors related to reproductive health and PCC behaviours in women of reproductive age with DM. The 48-item RHAB questionnaire has since been used in several studies designed to improve PCC behaviours in women with DM [25–28]. The abridged version used in this study comprised of the following constructs: Perceived susceptibility: Participants’ belief of their personal susceptibility to problems such as unplanned pregnancy and pregnancy-related complications. Perceived benefits: Participants’ opinion that a behaviour change, e.g. seeking PCC, will promote positive reproductive health and prevent an unplanned pregnancy. Perceived barriers: Assessment of obstacles that may deter a behaviour change, e.g. seeking PCC. Self-efficacy: Self-confidence to seek PCC and use contraception to prevent an unplanned pregnancy. Outcome expectations: Participants’ belief that outcomes are influenced by their own decisions to seek PCC.
3) Preconception care knowledge (K) instrument	Pre- and post-intervention questionnaire	At baseline and after 3 months of using the app	20 (True/False/Not sure) questions on knowledge of pregnancy planning (11 questions) and pregnancy-related risks (9 questions). The Knowledge (K) instrument [28] was adapted for use in women with DM in the United Kingdom from a validated instrument with acceptable levels of validity and reliability (Cronbach’s alpha = 0.65–0.83).
4) Patient activation measure (PAM)	Pre- and post-intervention questionnaire	At baseline and after 3 months of using the app	13-item (5 point Likert scale) patient activation measure (PAM) instrument, a widely used measure of activation and an important determinant of health behaviour / improved outcomes [70, 81]. The PAM is a validated instrument and the 13-item short form used in this study has good validity and internal reliability (Cronbach’s alpha = 0.84–0.89). The PAM is used to measure extent of behaviour change before and after an intervention. The total pre- and post-intervention PAM score was calculated by adding up the responses to the 13 questions. These raw scores were then transformed to a scale from 0-100, with the aid of a nomogram provided by Hibbard et al. and used to segment participants into one of four activation levels: Level 1 (< 47.0): Patients who do not feel in control of their own health and care. Level 2 (47.1–55.1): Patients who have low confidence in their ability to manage their health. Level 3 (55.2–67.0): Patients who have some experience and success in making behavioural changes. Level 4 (> 67.1): Patients who have made most of the behavioural changes and can maintain these over time.
5) App satisfaction	Post-intervention questionnaire	After 3 months of app use	Visual Analogue Scale used to assess overall satisfaction with the app and its functionalities; 0 = not satisfied and 100 = completely satisfied.

*DM* diabetes mellitus

*PCC* preconception care
